# Coronary heart disease risk factors in taxi drivers in Medellín

**DOI:** 10.1093/occmed/kqag065

**Published:** 2026-08-10

**Authors:** D M Gómez-Gallego, D E Rodríguez-Niño, J D González-Castaño, V Castañeda-Quintero, J A Callejas-Valencia, M Montoya Castillo, S Rendon-Marin, L F Higuita-Gutiérrez

**Affiliations:** Facultad de Medicina, Universidad Cooperativa de Colombia, sede Medellín, Medellín 050012, Colombia; Escuela de Microbiología, Universidad de Antioquia, Medellín 050010, Colombia; Escuela de Microbiología, Universidad de Antioquia, Medellín 050010, Colombia; Escuela de Microbiología, Universidad de Antioquia, Medellín 050010, Colombia; Escuela de Microbiología, Universidad de Antioquia, Medellín 050010, Colombia; Facultad de Medicina, Universidad Cooperativa de Colombia, sede Medellín, Medellín 050012, Colombia; Grupo de Investigaciones Biomédicas Uniremington, Facultad de Ciencias de la Salud, Programa de Medicina, Corporación Universitaria Remington, Medellín 050012, Colombia; Facultad de Medicina, Universidad Cooperativa de Colombia, sede Medellín, Medellín 050012, Colombia

## Abstract

**Background:**

Cardiovascular diseases are the leading cause of death worldwide. Taxi drivers constitute a high-risk occupational group due to prolonged sedentary work, extended shifts and chronic stress, beyond traditional cardiovascular risk factors.

**Aims:**

To describe the prevalence of coronary heart disease (CHD) risk factors and their sociodemographic and occupational associations among taxi drivers in Medellín, Colombia.

**Methods:**

We conducted a cross-sectional study of 484 taxi drivers recruited citywide. We obtained data using a structured questionnaire on sociodemographic and occupational factors and self-reported diagnoses of hypertension, dyslipidaemia and diabetes. We assessed physical activity with the International Physical Activity Questionnaire, and alcohol and tobacco use with the Alcohol, Smoking and Substance Involvement Screening Test. We estimated prevalences and analysed associations with multiple risk factors using chi-squared test and Poisson regression with a robust error variance.

**Results:**

Sedentary lifestyle prevalence was 71%; hypertension 30%; dyslipidaemia 24%; hazardous alcohol use 25%; hazardous tobacco use 14%; and diabetes 10%. Overall, 52% of participants had two or more CHD risk factors. In adjusted models, a higher prevalence of multiple risk factors was observed among drivers with primary education (adjusted prevalence ratio [aPR] = 1.71; 95% CI 1.26–2.32) and secondary education (aPR = 1.43; 95% CI 1.06–1.93), more than 10 years of driving experience (aPR = 1.39; 95% CI 1.09–1.75) and mixed day and night shifts (aPR = 1.18; 95% CI 1.01–1.40), after adjustment for sociodemographic and occupational variables.

**Conclusions:**

Taxi drivers in Medellín show a high burden of CHD risk factors, underscoring the need for targeted occupational health interventions addressing modifiable conditions.

Key learning points **What is already known about this subject:** 

- Taxi drivers are considered a high-risk occupational group due to long working hours, sedentary lifestyles and psychosocial stress.
- International studies have reported elevated rates of hypertension, diabetes and metabolic syndrome in professional drivers.
- However, evidence from Latin America remains scarce, limiting region-specific occupational health interventions.

**What this study adds:** 

- More than half of taxi drivers in Medellín had multiple risk factors for coronary heart disease.
- Lower educational attainment, longer years of employment and night or mixed shifts were independently associated with increased risk.
- Taxi driving itself contributes to cardiovascular vulnerability beyond traditional sociodemographic determinants.

**What impact this may have on practice or policy:** 

- Findings support recognition of taxi driving-related working conditions as occupational risk factors for cardiovascular disease.
- Preventive strategies in occupational health programmes should target modifiable behaviours, particularly physical inactivity, smoking and hazardous alcohol use.
- Policymakers should consider structural interventions such as shift regulation, health promotion and improved access to preventive care for drivers.

## Introduction

Cardiovascular diseases (CVDs) are the leading cause of mortality worldwide, accounting for an estimated 17.9 million deaths annually [1]. They impose substantial health and economic burdens, particularly in low- and middle-income countries [2]. Coronary heart disease (CHD), largely driven by atherosclerosis, remains a major contributor to morbidity and mortality. Established risk factors include hypertension, dyslipidaemia, diabetes mellitus, smoking, obesity, sedentary lifestyles and chronic stress [3].

Occupational conditions are increasingly recognized as determinants of cardiovascular health, particularly in jobs characterized by prolonged sitting, high psychological demands and environmental exposures [4,5]. Taxi driving exemplifies this profile, involving extended sedentary periods, irregular schedules, chronic occupational stress, limited opportunities for healthy behaviours and exposure to urban air pollution [6–8]. Evidence indicates elevated levels of stress, anxiety and psychological distress among taxi drivers, with reported stress prevalence ranging from 19 to 55%, associated with mental strain, poor working conditions, insufficient sleep and work–family conflict [6]. These psychosocial stressors may interact with sedentary work and other occupational exposures, contributing to clustering of cardiometabolic risk factors beyond traditional lifestyle determinants [9].

International studies consistently report elevated cardiovascular risk among professional drivers. High rates of CVD and diabetes linked to occupational factors have been observed in East Africa [10], while studies in Spain and South Africa report substantial burdens of cardiovascular risk factors and metabolic syndrome, associated with smoking, physical inactivity and longer driving careers [11,12]. Evidence from Iran and the USA similarly documents a high prevalence of overweight, hypertension and metabolic abnormalities related to extended working hours, low physical activity and mental fatigue [8,13,14].

Environmental exposures further compound risk: Studies in Brazil and China demonstrate associations between air pollutants and vascular and autonomic alterations among taxi drivers [7,15,16].

In Colombia, taxi driving operates within a regulated transport system; however, employment arrangements are heterogeneous and frequently informal. Many drivers work under revenue-based or leasing agreements (‘producido’), requiring daily income generation to meet fixed vehicle and living costs. In the Medellín metropolitan area, such arrangements are associated with extended working days, often exceeding 10 h, particularly among non-owner drivers [17]. National evidence also indicates a substantial cardiometabolic burden among Colombian professional drivers. Studies in Bogotá, Armenia and Manizales report high prevalences of hypertension, excess weight, metabolic syndrome and low physical activity among public service drivers [18–20].

Medellín, one of Colombia’s largest cities, has ∼19 000 licensed taxis, highlighting the relevance of this occupational group. Despite international and national evidence, no study has specifically examined cardiovascular risk factors among taxi drivers in this setting. Contextual factors related to urban infrastructure, labour organization and sociodemographic characteristics may shape distinct risk patterns. However, evidence generated in Medellín may also be informative for other Latin American cities with similar urban dynamics and occupational conditions, where data on cardiovascular risk among taxi drivers remain scarce. This lack of region-specific evidence limits the development of tailored occupational health interventions. Therefore, this study aimed to describe the prevalence of CHD risk factors and their sociodemographic and occupational associations among taxi drivers in Medellín, Colombia.

## Methods

We conducted a descriptive cross-sectional study among taxi drivers in Medellín. We calculated the sample size assuming an expected prevalence of high cardiovascular risk of 35% [13], a 95% confidence level and set precision at 5%, using Epidat version 3.1, a freely available epidemiological data analysis software, developed by the Pan American Health Organization and the Xunta de Galicia [21]. The minimum required sample was 346 drivers; we increased this by 15% to account for potential losses, resulting in a target of 398 participants.

We recruited participants using non-probabilistic, consecutive sampling at designated taxi stands (‘acopios’) across the northern, southern, eastern and western sectors of the city (Figure 1). Recruitment was conducted on different days and at varying times to capture diverse working patterns. Approximately 10% of eligible drivers declined participation, mainly due to time constraints. Eligible participants were male licensed taxi drivers aged ≥18 years, actively working in Medellín, with at least 1 year of driving experience, who provided written informed consent. We excluded drivers who declined participation or were unable to complete the questionnaire.

**Figure 1:**
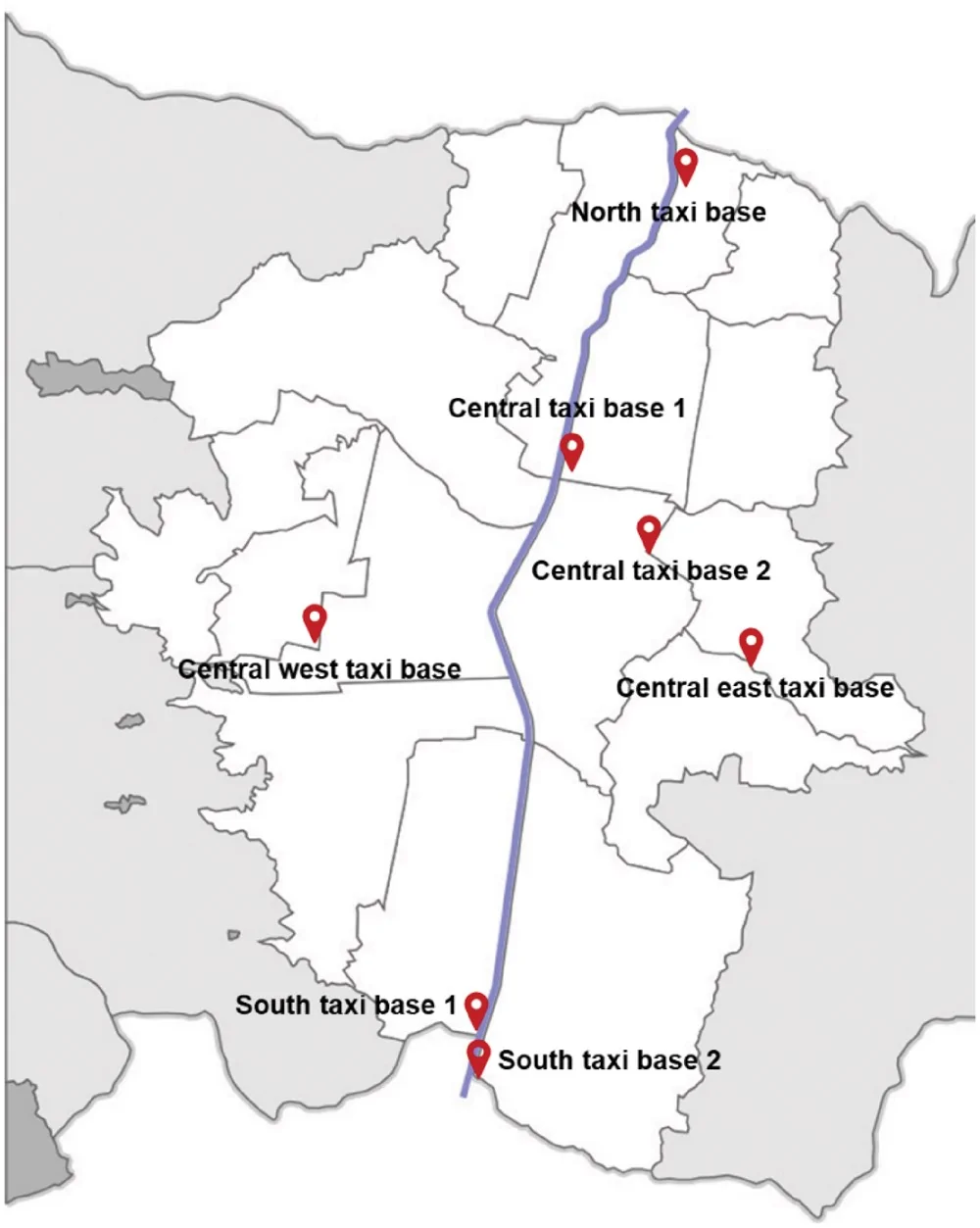
Geographic distribution of taxi driver recruitment sites in Medellín.

We collected data through a face-to-face interviewer-administered survey conducted during drivers’ working shifts while waiting for passengers. Interviews were performed by trained field staff and lasted ∼10–15 min. The questionnaire included sociodemographic and occupational characteristics and self-reported diagnoses of hypertension, dyslipidaemia and diabetes. We assessed physical activity using the International Physical Activity Questionnaire [22], calculating weekly energy expenditure in Metabolic Equivalent of Task (MET)-minutes and classifying participants as sedentary (<600 MET-min/week) or physically active (≥600 MET-min/week).

We evaluated risky alcohol and tobacco use using version 3.1 of the Alcohol, Smoking and Substance Involvement Screening Test (ASSIST), categorizing participants into standard WHO risk levels for each substance [23].

We summarized data using descriptive statistics with 95% confidence intervals (CIs). We estimated prevalences of individual risk factors and calculated prevalence ratios according to demographic and occupational characteristics. We compared groups using Pearson’s chi-squared test and the chi-squared test for linear trend, the latter applied to ordinal variables.

To identify factors associated with the presence of two or more CHD risk factors, we fitted Poisson regression models with a robust error variance [24]. The dependent variable was the presence of ≥2 risk factors (yes/no). Sociodemographic and occupational variables evaluated in the models included age group, education level, years working as a taxi driver, daily working hours and work shift. Variables were assessed as potential confounders based on epidemiological plausibility and bivariate analyses, and only those retaining an independent association with the outcome were included in the final multivariable model. We considered variables as potential confounders if they met the following criteria: (i) associated with the dependent variable in bivariate analysis, (ii) associated with another independent variable in bivariate analysis and (iii) their association with the dependent variable disappeared in multivariable analysis. We conducted all analyses using SPSS version 29, and *P* value <0.05 were considered statistically significant.

The Research Bioethics Committee of the Universidad Cooperativa de Colombia approved the study protocol (Bioethical Concept No. BIO360; Act No. 006, 1 June 2023). All participants provided informed consent, and we conducted the study in accordance with the Declaration of Helsinki and Colombian regulations.

## Results

We included 484 male taxi drivers with a median age of 55 years (interquartile range [IQR]: 45–62). Most were married (41%), had completed secondary education (55%), belonged to the middle socioeconomic stratum (46%) and were affiliated with the national social security health system (89%). Regarding occupational characteristics, 59% had worked as taxi drivers for more than 10 years, 59% worked every day of the week, the mean daily working time was 12 ± 2.6 h and 36% worked night shifts (Table 1).

**Table 1:** Demographic and occupational characteristics of taxi drivers included in the study

Variable	*n*	%	95% CI
**Age group**			
20–39 years	74	15	12.3–18.7
40–59 years	248	51	46.8–55.7
≥60 years	162	33	29.4–37.8
**Marital status**			
Single	170	35	31.0–39.5
Cohabiting	118	24	20.7–28.4
Married	196	41	36.2–44.9
**Socioeconomic status**			
Low	247	51	46.6–55.5
Middle	224	46	41.9–50.7
High	13	3	1.5–4.4
**Education**			
Primary	128	26	22.7–30.5
Secondary	266	55	50.5–59.4
Technical or higher	90	19	15.3–22.2
**Health insurance affiliation**			
Yes	416	86	82.6–88.8
No	68	14	11.2–17.4
**Years working as taxi driver**			
1–5 years	111	24	19.8–27.4
6–10 years	84	18	14.5–21.4
>10 years	278	59	54.3–63.1
**Daily working hours**			
1–8 h	53	11	8.5–14.2
9–12 h	231	48	44.0–52.9
>12 h	193	41	36.1–44.9
**Work shift**			
Daytime only	303	64	59.4–68.0
Daytime and nighttime	172	36	32.0–40.6

The prevalence of CHD risk factors was sedentary lifestyle 71% (95% CI 67.1–75.2), hypertension 30% (95% CI 25.6–33.7), dyslipidaemia 24% (95% CI 20.3–27.9), hazardous alcohol use 25% (95% CI 21.1–28.8), hazardous tobacco use 15% (95% CI 11.5–17.8) and diabetes 10% (95% CI 7.9–13.3). Overall, 52% (95% CI 47.2–56.1) presented two or more CHD risk factors (Figure 2); the median number per driver was 2 (IQR: 1–2).

**Figure 2:**
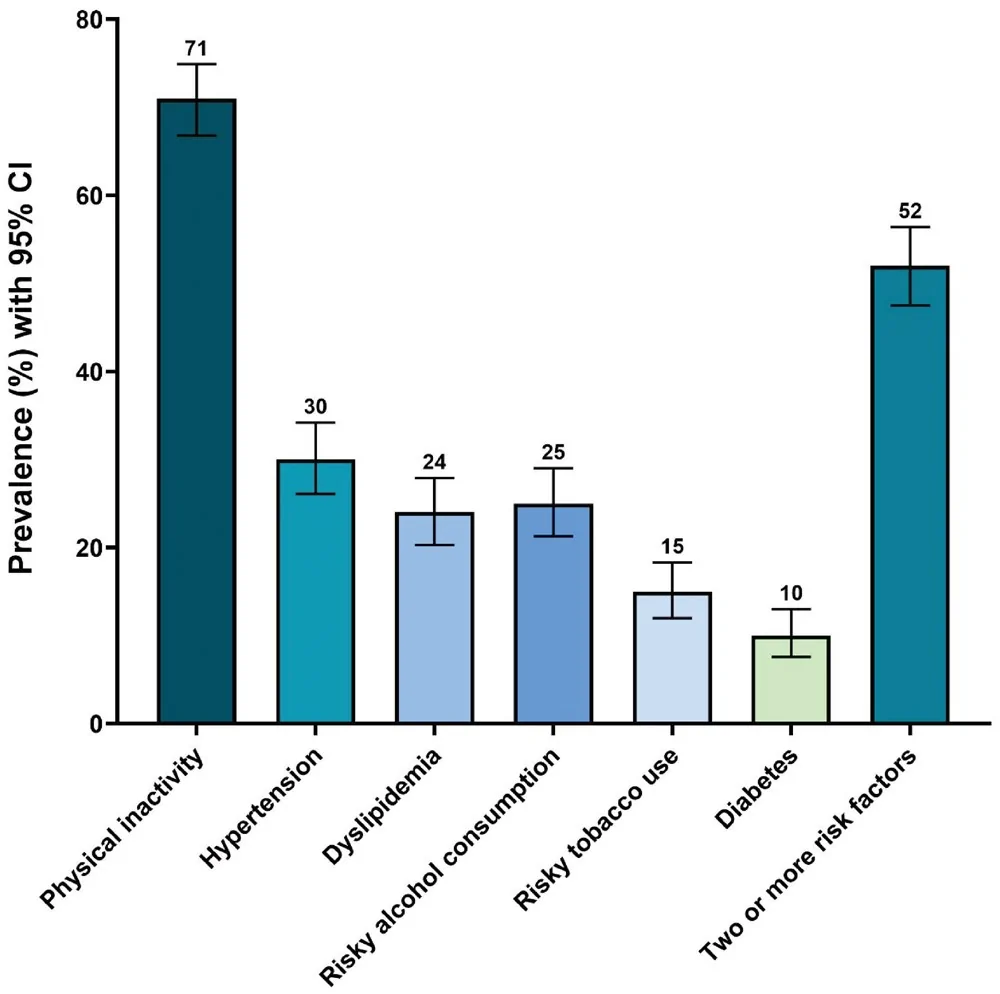
Prevalence of risk factors for coronary heart disease in taxi drivers, Medellín.

To identify groups with multiple CHD risk factors, we compared demographic and occupational characteristics between drivers with two or more risk factors and those with fewer. The prevalence increased with age (*P* < 0.05), decreased with higher educational attainment (*P* < 0.001) and was higher among those with more years working as taxi drivers and longer daily working hours (*P* < 0.05) (Table 2).

**Table 2:** Prevalence ratios for multiple coronary heart disease risk factors according to demographic and occupational characteristics of taxi drivers

Variable	<2 Risk factors		≥2 Risks factors		Crude prevalence ratio	95% CI	*P* value
	*N*	%	*n*	%			
**Age group**							* ^a^
20–39 years	41	55	33	45	1.00	Reference	
40–59 years	125	50	123	50	1.13	0.85–1.51	
≥60 years	68	42	94	58	1.31	0.98–1.76	
**Marital status**							n.s ^b^
Single	81	48	89	52	1.00	Reference	
Cohabiting	64	54	54	46	0.90	0.71–1.15	
Married	89	45	107	55	1.05	0.87–1.28	
**Socioeconomic status**							n.s ^a^
Low	122	49	125	51	1.00	Reference	
Middle	106	47	118	53	1.03	0.86–1.22	
High	6	46	7	54	1.03	0.62–1.73	
**Health insurance**							n.s ^b^
No	36	53	32	47	1.00	Reference	
Yes	198	48	218	52	1.12	0.86–1.47	
**Education**							*** ^a^
Primary	46	36	82	64	1.70	1.26–2.28	
Secondary	132	50	134	50	1.33	0.99–1.78	
Technical or higher	56	62	34	38	1.00	Reference	
**Years working as taxi driver**							** ^a^
1–5 years	65	59	46	41	1.00	Reference	
6–10 years	45	54	39	46	1.10	0.80–1.51	
>10 years	116	42	162	58	1.40	1.10–1.78	
**Daily working hours**							* ^a^
1–8 h	32	60	21	40	1.00	Reference	
9–12 h	112	49	119	52	1.30	0.91–1.85	
>12 h	85	44	108	56	1.41	0.99–2.01	
**Work shift**							n.s^b^
Daytime only	153	51	150	50	1.00	Reference	
Day and night	75	44	97	56	1.13	0.95–1.35	

a Chi-squared test for linear trend.

b Pearson’s chi-squared test.

* *P* < 0.05.

** *P* < 0.01.

*** *P* < 0.001.

n.s, not significant.

In multivariable Poisson regression, lower education, longer driving experience and mixed work shifts remained independently associated with the presence of ≥2 risk factors (Table 3). Primary education was associated with a 71% higher prevalence (adjusted prevalence ratio [aPR] = 1.71; 95% CI 1.26–2.32; *P* = 0.001), and secondary education with a 43% higher prevalence (aPR = 1.43; 95% CI 1.06–1.93; *P* < 0.05), compared with technical or higher education. Drivers with more than 10 years of experience had a 39% higher prevalence (aPR = 1.39; 95% CI 1.09–1.75; *P* < 0.01) than those with 1–5 years. Working both day and night shifts was associated with an 18% higher prevalence compared with daytime-only shifts (aPR = 1.18; 95% CI 1.01–1.40; *P* < 0.05).

**Table 3:** Poisson regression model with robust variance for occupational and educational characteristics associated with multiple cardiovascular risk factors among taxi drivers

Variables in the model^a^	Adjusted prevalence ratio	95% CI	*P* value
**Education**			**
Primary versus technical/higher	1.71	1.26–2.32	***
Secondary versus technical/higher	1.43	1.06–1.93	*
**Years working as taxi driver**			*
>10 years versus 1–5 years	1.39	1.09–1.75	**
6–10 years versus 1–5 years	1.13	0.83–1.54	n.s
**Work shift**			
Day + night versus day only	1.18	1.01–1.40	*

a The initial multivariable Poisson regression model included age group, education level, years working as a taxi driver, daily working hours and work shift. Age group and daily working hours were not retained in the final model due to lack of independent association after adjustment. The table shows adjusted prevalence ratios for variables included in the final model.

* *P* < 0.05.

** *P* < 0.01.

*** *P* < 0.001.

## Discussion

This study identified a high prevalence of CHD risk factors among taxi drivers in Medellín, particularly sedentary behaviour (71%) and hypertension (30%). The prevalence of dyslipidaemia (24%) and diabetes (10%) was also substantial and aligns with reports from other settings linking these conditions to prolonged sedentary work, chronic stress and limited access to preventive health [8,10,12,14,18].

Hazardous alcohol use (25%) was comparable to findings in similar populations; for example, 31% of professional drivers in New Delhi reported consuming alcohol at least twice weekly [25]. Alcohol misuse is associated with chronic disease and reduced quality of life [4,23], and occupational stress—arising not only from long working hours but also from broader psychosocial demands—may contribute to alcohol use as a coping strategy [26]. Tobacco risk consumption (15%) was consistent with previous Colombian studies among transport drivers [27–29], although differences across studies may reflect variations in measurement instruments and population characteristics.

More than half of participants (52%) exhibited multiple risk factors. Lower educational attainment, longer driving careers and mixing day–night shifts were independently associated with this clustering. Drivers with primary or secondary education had a markedly higher prevalence of multiple risk factors compared with those holding technical or higher qualifications, reinforcing the role of socioeconomic inequalities in shaping cardiovascular outcomes [30]. Prolonged occupational exposure may contribute through cumulative stress and sedentary behaviour [31], while mixed shifts disrupt circadian rhythms and metabolic homeostasis [32]. Moreover, evidence from studies conducted in the USA has shown that extended work shifts among taxi drivers are associated with adverse haematological and cardiovascular markers, supporting the biological plausibility of the associations observed in our study [13,18]. Together, these findings highlight the intersecting influence of inequities in education, the intensity of occupational exposures and atypical work schedules on cardiovascular vulnerability in taxi drivers.

These findings must be interpreted within the specific labour context of taxi driving in Medellín. Many drivers operate under informal or semi-formal revenue-based arrangements (‘producido’), relying on daily earnings to cover fixed vehicle payments and living costs. Evidence from the Valle de Aburrá indicates that extended working days—often exceeding 10 h—are common and frequently driven by economic pressure rather than choice, particularly among drivers who do not own their vehicles [17]. Such structural conditions constrain autonomy over working hours and may intensify cumulative exposure to cardiovascular risk factors.

International evidence supports this pattern. Studies in South Africa and Iran have reported high prevalences of metabolic syndrome, hypertension, overweight and clustered risk factors among taxi drivers, associated with longer employment duration, lower education and poor sleep quality [12,14]. Similar findings have been documented in Latin America. In Bogotá and Armenia (Colombia), public transport drivers exhibited high cardiometabolic risk profiles [18,19], while Peruvian and Mexican studies have described associations between sociodemographic characteristics, working conditions and cardiovascular risk among taxi drivers [33,34]. Collectively, these data suggest that the cardiovascular burden observed in Medellín reflects broader occupational and structural determinants affecting urban transport workers in comparable settings.

These findings underscore the need for targeted occupational health strategies. Periodic cardiovascular screening integrated into occupational health services, health promotion initiatives addressing physical activity, diet, smoking cessation, alcohol reduction and stress management, and regulatory measures to limit excessive working hours and prolonged night shifts represent feasible interventions. Effective action will likely require collaboration between health authorities, transport regulators and taxi associations to address both behavioural and structural determinants.

Several limitations should be acknowledged. The cross-sectional design precludes causal inference. The use of convenience sampling, the exclusion of women and digital platform drivers and potential selection bias limit generalizability. Cardiovascular risk factors were based on self-reported diagnoses, which may have led to underestimation due to undiagnosed conditions or social desirability bias in reporting alcohol and tobacco use. Nevertheless, self-reported measures are widely used in occupational epidemiology when clinical assessment is impractical, and previous research supports their utility, although sensitivity varies across conditions [35,36].

Although validated cardiovascular risk scores exist [37,38], applying them was not feasible given logistical constraints, limited time availability and the mobility inherent to taxi driving [39]. Extended working hours—particularly among those exceeding 12 h per day—require special consideration. While Colombian regulations govern working time in the transport sector [40], enforcement may be inconsistent, especially under informal revenue-based arrangements [17], potentially increasing cumulative exposure to occupational risk [4,41]. Residual confounding cannot be excluded, as dietary patterns, psychosocial stress and sleep quality were not directly measured.

Despite these limitations, this study provides robust evidence of a substantial burden of CHD risk among taxi drivers in Medellín. Sedentary behaviour, hypertension, dyslipidaemia, hazardous alcohol and tobacco use, and diabetes were common, and risk clustering increased with longer careers and mixed shifts. Importantly, cardiovascular vulnerability appears linked not to taxi driving per se, but to modifiable features of work organization, including prolonged working hours, extended occupational exposure, irregular shifts and constrained opportunities for healthy behaviours. Addressing these structural and behavioural determinants offers a realistic pathway to reducing cardiovascular risk among taxi drivers and informing sustainable occupational health policies in Medellín and similar urban contexts.
